# Supplementary material for: Transient histone deacetylase inhibition induces cellular memory of gene expression and 3D genome folding
Source: Nat Genet. 2026 Feb 4;58(2):404–17. doi: 10.1038/s41588-025-02489-4 (PMC12900649; doi:10.1038/s41588-025-02489-4)
Supplement: Supplementary file 1 — Supplementary Methods. [file 41588_2025_2489_MOESM1_ESM.pdf]

nature genetics

Supplementary information

https://doi.org/10.1038/s41588-025-02489-4

Transient histone deacetylase inhibition induces cellular memory of gene expression and 3D genome folding

In the format provided by the authors and unedited

Supplementary methods

qPCR primer sequences (5'-3')

ActB-pos
F- GCTCATCAAATGCCCACACC
R- GCGTGTAGACTCTTTGCAGC

Oct4-pos
F- GAGGCAGCAGTGAAGGGAAT
R- CCAGGCCGGTTTCTGATTCT

Oct4-neg
F- ACAAGTCTTCAGCTCTGGCC
R- CCTGCCTCACCGGATAGTTG

Zfp608-neg
F-CAAATAGCTCTGACTCCTTAACC
R- GGACATTTACTTTTGTTGCCAATAG

Biophysical modelling

Two polymeric systems were prepared in 30 and 5 replicates respectively with 1 and 20 chains of 20 mega-basepairs (Mbp) each. Each chain-bead was of unitary mass (m = 1.0), hosting $\nu = 5$ kilo-base pairs (kbp) of DNA sequence and has a diameter of σ. This representation was obtained with the Kremer-Grest bead-spring model[1] with the same parameter as in[2].

$$H = U_{EV} + U_C + U_{BEND} \quad (1)$$

The first term was a truncated and shifted Lennard-Jones potential that controls the *cis*- and *trans*-chromosome excluded volume interactions:

$$U_{EV}(i,j) = \begin{cases} 4k_BT\,\epsilon_{ij}\left[\left(\frac{\sigma}{d_{i,j}}\right)^{12} - \left(\frac{\sigma}{d_{i,j}}\right)^{6} + {}^{1}/_{4}\right] & if\ d_{i,j} \le 2^{1/6}\sigma, \\ 0 & if\ d_{i,j} > 2^{1/6}\sigma. \end{cases} \quad (2)$$

where k_B is the Boltzmann constant, T the temperature, ϵ_{ij} is equal to 10 if $|i - j| = 1$, and 1 otherwise, σ was the thickness of the chain and $d_{i,j}$ is the modulus of $\overrightarrow{d_{ij}} = \overrightarrow{r_i} - \overrightarrow{r_j}$, that is the distance vector between the monomers *i* and *j* at positions $\overrightarrow{r_i}$ and $\overrightarrow{r_j}$, respectively.

The second term was a FENE potential that maintains chain connectivity between consecutive beads on the same polymer chain:

$$U_C(i, i+1) = -0.5KR_0^2 ln[1 - \left(\frac{d_{i,i+1}}{R_0}\right)^2 \quad (3)$$

where K=0.33 k_BT/nm^2 and R_0=1.5σ. The combined action of the connectivity and excluded volume interaction between consecutive beads was such that the average bond length was close to σ and never exceeded 1.1σ.

The third term is a (Kratky-Porod) bending potential:

$$U_{BEND} = \frac{k_B T K_\theta}{\sigma}\left(1 - \frac{d_{i,i+1} \cdot d_{i+1,i+2j}}{d_{i,i+1} d_{i+1,i+2j}}\right) \quad (4)$$

where K_θ is the chain persistence length.

The dynamics of the polymer model was simulated using the LAMMPS simulation package (version 29 Oct 2020) integrating the (underdamped) Langevin equation of motion[3]:

$$m\ddot{r}_{i\alpha} = -\partial_{i\alpha} H - \gamma \dot{r}_{i\alpha} + \eta_{i\alpha}(t) \quad (5)$$

where m is the mass of the bead that was set equal to the LAMMPS default value, H is the Hamiltonian of the system in Eq. (1), the index *i* runs over all the particles in the system, and $\alpha = (x, y, z)$ indicates the Cartesian components, and $\gamma = 0.5\ \tau_{LJ}^{-1}$ is the friction coefficient with $\tau_{LJ} = \sigma(m/\varepsilon)^{1/2}$ is the Lennard-Jones time. The stochastic term $\eta_{i\alpha}$ satisfies the fluctuation-dissipation conditions. The integration time step used in the numerical integration was equal to $\Delta t = \alpha\ \tau_{LJ}$, where the factor α was adapted, as specified below, to the different stages of the preparation and production runs.

Preparation of the initial conformations. Each chain is initially organized in a rod-like folding featuring rosettes along the main axis and placed in random positions inside a confining sphere of radius R^* so to set up the volume density of the system to 3% (R^*= 25.5σ for the 1-chain system and R^*=69.3σ for the 20-chain system), avoiding clashes with other chains. The confining sphere is completely contained in a cubic simulation box with fixed boundary conditions. After an energy minimization (*LAMMPS command*: minimize 1.0e-4 1.0e-6 100000 100000), each of the polymeric system is compressed to reach the DNA density of 10%. These conditions were achieved by minimization (*LAMMPS command*: minimize 1.0e-4 1.0e-6 100000 100000) followed by molecular dynamics simulations of 600 τ_{LJ} (100,000 Δt with Δt = 0.006 τ_{LJ}) during which the radius of confining spheres is reduced from the minimum radius to include all the particles of the chains at time 0 to the target radius R (R= 17.1σ for the 1-chain system and R=46.4σ for the 20-chain system). At the target volume density of 10%, the polymer chains have parameters σ~54.2nm and K_θ ~92.3nm. These estimates were done by considering a fine-scale chromatin model with $\nu_{FS} = 100\ bp$, $\sigma_{FS} = 20\ nm$ and $K_{\theta FS} = 50\ nm$ and the coarse-grain procedure in[4]. Finally, each polymeric system is relaxed with molecular dynamics run of 30,600 τ_{LJ} (5,100,000 Δt with Δt = 0.006 τ_{LJ}). By comparing the average monomer Mean-Squared Displacement (MSD) in these relaxation runs and the MSD of non-transcribed genes measured[5] by live-cell imaging, we obtained an approximated estimate of the simulated time (in τ_{LJ}) corresponding to 1s ~ 9 τ_{LJ}. These conformations are next used as the initial conformations for the downstream simulations.

A/B compartmentalisation. To model the A/B compartmentalisation in the DMSO condition short-range interactions were used to test the attractions between the model regions which

correspond to A/B compartments. As shown in Fig. 2d, the first and the last 1 Mb were assigned to telomeric region and 6 blocks of A/B domains each of 1.5 Mb were defined in the central part. These interactions have been modelled using attractive Lennard-Jones potentials (see **Equation 2**) with cutoff=2.5σ, that allowed to include the attractive part of the Lennard-Jones potential. To allow efficient parameter sampling, we performed 4h simulations (21,600,000 Δt with $\Delta t = 0.006\ \tau_{LJ}$) for just one chain by varying the strengths of compartments' interactions (E_{AA} and E_{BB}) were varied in the range 0.00-0.40 k_BT in the 1-chain system with 4h trajectories (21,600,000 Δt with $\Delta t = 0.006\ \tau_{LJ}$). To infer these energies, A/B compartment-strengths at 10kb from the micro-C maps were matched against the correspondent quantities computed on the model chains[6]. The compartment strength (CS) profile is obtained by partitioning the A- (B-) domains in 150 bins and by averaging within each of them the CS per 10kb-bin of the Micro-C or models' contact-maps. The distance-cutoff for detecting contacts in the models was set to 150 nm~3beads. The Euclidean distance between A and B profiles was used to define compartment-specific ranks r_A and r_B ranging from 1 to max(r) where 1 is the best match with the experiments. A unique rank r was defined from the average of r_A and r_B. Finally, the similarity score was defined as (r-max(r))/(min(r)-max(r)) and it is equal to 1 for the condition that best describes the experimental CS-profile and to 0 for the least accurate one. This procedure resulted in the optimal values E_{AA}=0.080 and E_{BB}=0.00. Next, the DMSO and TSA condition were modelled by the system made of 20 chains simulated for with 4h-trajectories (21,600,000 Δt with $\Delta t = 0.006\ \tau_{LJ}$). The optimized A/B-compartment attractions (ε_{AA}=0.080 and ε_{BB}=0.00) were maintained and the bending rigidity of the A- and B-compartment domains were differentially increased. We explored several combinations varying $K_{\theta A}$ between 1 and 19 times and $K_{\theta B}$ between 0 and 4 times the nominal persistence length of K_θ ~92.3nm. The chromosome-averaged *trans*-contact ratios (TR) were computed by averaging the trans-contact ratio of 10kb-bins in each chromosome. The median was obtained on the distribution of these chromosome-averaged values. The absolute difference between the median values TR for all, only A and only B domains in the micro-C datasets and the models was used to define three ranks per each parameter set. A similarity score was defined from these three ranks applying a strategy analogous to one described above for CS profiles. Optimal values were $(K_{\theta A}, K_{\theta B}) = (1,0)K_\theta$ in DMSO and $(K_{\theta A}, K_{\theta B}) = (14,3)K_\theta$ in TSA. Model snapshots in Extended Data Fig. 2j were prepared using VMD[7].

1. Kremer, K. & Grest, G. S. Dynamics of entangled linear polymer melts: A molecular-dynamics simulation. *J. Chem. Phys.* **92**, 5057–5086 (1990).
2. Di Stefano, M., Paulsen, J., Lien, T. G., Hovig, E. & Micheletti, C. Hi-C-constrained physical models of human chromosomes recover functionally-related properties of genome organization. *Sci. Rep.* **6**, 1–12 (2016).

3. Plimpton, S. Fast Parallel Algorithms for Short-Range Molecular Dynamics. *J Comput. Phys.* **117**, 1–19 (1995).

4. Ghosh, S. K. & Jost, D. How epigenome drives chromatin folding and dynamics, insights from efficient coarse-grained models of chromosomes. *PLoS Comput. Biol.* **14**, 1–26 (2018).

5. Gu, B. *et al.* Transcription-coupled changes in nuclear mobility of mammalian cis-regulatory elements. *Science* **359**, 1050–1055 (2018).

6. Di Stefano, M., Nützmann, H. W., Marti-Renom, M. A. & Jost, D. Polymer modelling unveils the roles of heterochromatin and nucleolar organizing regions in shaping 3D genome organization in Arabidopsis thaliana. *Nucleic Acids Res.* **49**, 1840–1858 (2021).

7. Humphrey, W., Dalke, A. & Schulten, K. VMD: Visual molecular dynamics. *J. Mol. Graph.* **14**, 33–38 (1996).
