# Supplementary material for: Transient histone deacetylase inhibition induces cellular memory of gene expression and 3D genome folding
Source: Nat Genet. 2026 Feb 4;58(2):404–17. doi: 10.1038/s41588-025-02489-4 (PMC12900649; doi:10.1038/s41588-025-02489-4)
Supplement: Supplementary file 2 — Reporting Summary [file 41588_2025_2489_MOESM2_ESM.pdf]

nature portfolio

Corresponding author(s): Dr. Giacomo Cavalli

Last updated by author(s): 19/11/2025

Reporting Summary

Nature Portfolio wishes to improve the reproducibility of the work that we publish. This form provides structure for consistency and transparency in reporting. For further information on Nature Portfolio policies, see our Editorial Policies and the Editorial Policy Checklist.

Statistics

For all statistical analyses, confirm that the following items are present in the figure legend, table legend, main text, or Methods section.

n/a	Confirmed	
☐	☒	The exact sample size (n) for each experimental group/condition, given as a discrete number and unit of measurement
☐	☒	A statement on whether measurements were taken from distinct samples or whether the same sample was measured repeatedly
☐	☒	The statistical test(s) used AND whether they are one- or two-sided<br>*Only common tests should be described solely by name; describe more complex techniques in the Methods section.*
☒	☐	A description of all covariates tested
☐	☒	A description of any assumptions or corrections, such as tests of normality and adjustment for multiple comparisons
☐	☒	A full description of the statistical parameters including central tendency (e.g. means) or other basic estimates (e.g. regression coefficient) AND variation (e.g. standard deviation) or associated estimates of uncertainty (e.g. confidence intervals)
☐	☒	For null hypothesis testing, the test statistic (e.g. F, t, r) with confidence intervals, effect sizes, degrees of freedom and P value noted<br>*Give P values as exact values whenever suitable.*
☒	☐	For Bayesian analysis, information on the choice of priors and Markov chain Monte Carlo settings
☐	☒	For hierarchical and complex designs, identification of the appropriate level for tests and full reporting of outcomes
☒	☐	Estimates of effect sizes (e.g. Cohen's d, Pearson's r), indicating how they were calculated

Our web collection on statistics for biologists contains articles on many of the points above.

Software and code

Policy information about availability of computer code

Data collection	Zeiss ZEN Blue (v3.8–3.12)<br>CytExpert (v2.4)<br>NovoExpress (v1.6.3)<br>ChemiDoc Imaging Systems (BioRad)
Data analysis	Data analysis was performed as described in the Methods of the paper. Code for the novel analyses was deposited at https://github.com/cavallifly/Paldi_et_al_2024 and at https://doi.org/10.5281/zenodo.17608120<br><br>Softwares used in this study:<br>Subread (v2.0.6)<br>DEseq2 (1.42.1)<br>clusterProfiler (v4.10.1)<br>EnhancedVolcano (v1.20.0)<br>ggplot2 (v3.5.1)<br>HOMER (v4.10.0)<br>bowtie2 (v2.4.4)<br>samtools (v1.9)<br>Sambamba (v1.0)<br>IGV (v.2.16.1)<br>HiGlass (v1.11.8)<br>MACS3 (v3.0.3)

diffBind (v3.12.0)
deepTools (v3.5.6)
ChIPseeker (v1.38.0)
TrimGalore (v0.6.10)
HiC-Pro (v3.1.0)
HiCRep (v1.12)
cooltools (v0.5.4)
mustache (v1.0)
bedtools (v2.31.1)
coolpuppy (v1.1.0)
Imaris (v10.1.1)
FlowJo (v10.10)
LightCycler (v1.5.1)
Fiji (v2.14.0)

For manuscripts utilizing custom algorithms or software that are central to the research but not yet described in published literature, software must be made available to editors and reviewers. We strongly encourage code deposition in a community repository (e.g. GitHub). See the Nature Portfolio guidelines for submitting code & software for further information.

Data

Policy information about availability of data

All manuscripts must include a data availability statement. This statement should provide the following information, where applicable:

- Accession codes, unique identifiers, or web links for publicly available datasets
- A description of any restrictions on data availability
- For clinical datasets or third party data, please ensure that the statement adheres to our policy

All raw data were submitted to the National Library of Medicine's (NCBI) Sequence Read Archive (SRA) and processed files were submitted to Gene Expression Omnibus (GEO). All data can be retrieved under the GEO series GSE281151. Myc ChIP-seq dataset was published was downloaded from the GEO repository GSE90895. NPC H3K27me3 ChIP-seq was downloaded from the GEO repository GSE262551.

Research involving human participants, their data, or biological material

Policy information about studies with human participants or human data. See also policy information about sex, gender (identity/presentation), and sexual orientation and race, ethnicity and racism.

Reporting on sex and gender	N/A
Reporting on race, ethnicity, or other socially relevant groupings	N/A
Population characteristics	N/A
Recruitment	N/A
Ethics oversight	N/A

Note that full information on the approval of the study protocol must also be provided in the manuscript.

Field-specific reporting

Please select the one below that is the best fit for your research. If you are not sure, read the appropriate sections before making your selection.

☒ Life sciences ☐ Behavioural & social sciences ☐ Ecological, evolutionary & environmental sciences

For a reference copy of the document with all sections, see nature.com/documents/nr-reporting-summary-flat.pdf

Life sciences study design

All studies must disclose on these points even when the disclosure is negative.

Sample size	No statistical methods were used to determine sample sizes. Instead, they were defined in compliance with gold standards in the field. Micro-C experiments were performed in 1 million cells. RNA-seq samples were isolated from a number of cells varying between 200 000 and 1 million. ChIP-seq and ChIP-qPCR were performed on approximately 4 million cells for TFs and 2 million cells for histone marks. Immunostainings were performed on ~10 gastruloids per replicate. ~100 cells per condition were imaged for ESC immunofluorescence. ATAC-seq were performed on 100 000 cells. Western blots were produced with protein extracts from ~10^7 cells. ~20000 cells per condition were assayed by FACS.
Data exclusions	For RNA-seq analysis any gene with less than 10 reads was excluded from analysis because of insufficient statistical power in the downstream

Data exclusions	analyses. For immunofluorescence, gastruloids with abnormal symmetry were not chosen for imaging.
Replication	Micro-C experiments were performed in 5 (DMSO, TSA), 2 (24h recovery, Romidepsin) or 1 (PCGF2 -OHT +DMSO, +OHT +DMSO, +OHT +TSA, +OHT +REC) biological replicates. RNA-seq and gastruloid experiments were performed in biological triplicates. ChIP-seq, ChIP-qPCR and ATAC-seq were performed in biological duplicates except for H3K27ac ChIP-seq (3 biological replicates). ESC immunofluorescence was performed in biological duplicates. For sequencing-based experiments PCA and Spearman correlation was routinely performed to assess reproducibility. Reproducibility of Micro-C experiments was assessed using the Stratum-adjusted Correlation Coefficient from the HiCRep package.
Randomization	This study does not require randomization protocols.
Blinding	Blinding is not compatible with this study as the identity of control samples must be known for genomic data analyses. Data reported here are based on unbiased analysis.

Reporting for specific materials, systems and methods

We require information from authors about some types of materials, experimental systems and methods used in many studies. Here, indicate whether each material, system or method listed is relevant to your study. If you are not sure if a list item applies to your research, read the appropriate section before selecting a response.

Materials & experimental systems

n/a	Involved in the study
☐	☒ Antibodies
☐	☒ Eukaryotic cell lines
☒	☐ Palaeontology and archaeology
☒	☐ Animals and other organisms
☒	☐ Clinical data
☒	☐ Dual use research of concern
☒	☐ Plants

Methods

n/a	Involved in the study
☐	☒ ChIP-seq
☐	☒ Flow cytometry
☒	☐ MRI-based neuroimaging

Antibodies

Antibodies used

The following antibodies were used (from multiple lots over the course of the study):
- H3K4me1 (ActiveMotif #39297) - Wb: 1:5000, ChIP: 3µl
- H3K4me3 (Millipore #04-745) - Wb: 1:1000, ChIP: 3µl
- H3K9me3 (abcam #8898) - Wb: 1:2000, ChIP: 3µl
- H3K27me3 (ActiveMotif #39155) - Wb: 1:2500, ChIP: 3µl
- H3K27Ac (ActiveMotif #39133) - Wb: 1:7500, ChIP: 3µl
- H2AK119Ub (Cell Signalling #8240S) - Wb: 1:2000, ChIP: 3µl
- CTCF (Active Motif #61311) - ChIP: 5µl
- YY1 (abcam #109237) - ChIP: 8µl
- Pan-Acetyl-Lysine (Proteintech #66289-1-IG) - Wb: 1:1000,
- H3K9Ac (Millipore #07-352) - Wb: 1:7500, ChIP: 3µl
- Ring1B (Cell Signalling #5694) - ChIP: 5µl
- Acetyl-Tubulin (Sigma-Aldrich #T7451) - Wb: 1:2000
- Lamin B1 (abcam #ab16048) - Wb: 1:10000

Validation

The antibodies were validated by the manufacturer as follows:
- H3K4me1 (ActiveMotif #39297) "Applications Validated by Active Motif: ChIP: 5 - 10 µg per ChIP WB*: 0.2 - 2 µg/ml dilution DB: 1 µg/ml dilution." All other information can be found at https://www.activemotif.com/catalog/details/61781/histone-h3k4me1-antibody-pab-4.
- H3K4me3 (Millipore #04-745) "Anti-trimethyl-Histone H3 (Lys4) Antibody, clone MC315 is a rabbit monoclonal antibody for detection of trimethyl-Histone H3 (Lys4) also known as H3K4me3, Histone H3 (tri methyl K4) & has been validated in WB, ChIP, DB, Mplex, ChIP-seq." All other information can be found at https://www.merckmillipore.com/FR/fr/product/Anti-trimethyl-Histone-H3-Lys4-Antibody-clone-MC315-rabbit-monoclonal,MM_NF-04-745.
- H3K9me3 (abcam #8898) "Every new batch of ab8898 is tested in house in ChIP." All other information can be found at https://www.abcam.com/en-us/products/primary-antibodies/histone-h3-tri-methyl-k9-antibody-chip-grade-ab8898.
- H3K27me3 (ActiveMotif #39155) "Applications Validated by Active Motif: ChIP: 5 - 10 µg per ChIP ChIP-Seq: 5 µg each ICC/IF: 2 µg/ml dilution IHC(FFPE): 2 µg/ml dilution WB*: 0.5 - 2 µg/ml dilution CUT&Tag: 1 µg per 50 µl reaction* CUT&RUN: 1 µg per 50 µl reaction" All other information can be found at https://www.activemotif.com/catalog/details/39155.
- H3K27Ac (ActiveMotif #39133) "Validated by ActiveMotif for: ChIP: 10 µg per ChIP, ChIP-Seq: 5 µg each, ICC/IF: 1 - 5 µg/ml dilution, WB*: 0.1 - 1 µg/ml dilution, CUT&Tag: 1 µg per 50 µl reaction." All other information can be found at https://www.activemotif.com/catalog/details/39133/histone-h3-acetyl-lys27-antibody-pab.
- H2AK119Ub (Cell Signalling #8240S) "This antibody has been validated using SimpleChIP® Enzymatic Chromatin IP Kits." All other information can be found at https://www.cellsignal.com/products/primary-antibodies/ubiquityl-histone-h2a-lys119-d27c4-rabbit-monoclonal-antibody/8240.

-CTCF (Active Motif #61311) "Validated for: ChIP: 2 - 8 µl per ChIP,ChIP-Seq: 4 µg per ChIP, ICC/IF: 1:2,000 dilution, WB: 1:500-1:2,000 dilution, IHC(FFPE): 1:1000 dilution, CUT&Tag* 1 µl per 50 µl reaction, CUT&RUN: 1 µl per 50 µl reaction" All other information can found at https://www.activemotif.jp/documents/tds/61311.pdf
-YY1 (abcam #109237) Previously used in Dong et al. (2022) doi: 10.1093/nar/gkac230. All other information can be found at https://www.abcam.com/en-us/products/primary-antibodies/yy1-antibody-epr4652-nuclear-loading-control-ab109237.
-Pan-Acetyl-Lysine (Proteintech #66289-1-IG) "Tested Applications: WB, IF/ICC, ELISA; RecommendedDilutions: WB 1:500-1:3000 IF/ICC 1:50-1:500" All other information can be found at https://www.ptglab.com/fr/products/Pan-Acetylation-Antibody-66289-1-Ig.htm.
-H3K9Ac (Millipore #07-352) "Anti-acetyl-Histone H3 (Lys9) Antibody is a Rabbit Polyclonal Antibody for detection of acetyl-Histone H3 (Lys9) also known as H3K9Ac, Histone H3 (acetyl K9) and has been published and validated in ChIP, WB, Mplex." All other information can be found at https://www.merckmillipore.com/FR/fr/product/Anti-acetyl-Histone-H3-Lys9-Antibody,MM_NF-07-352.
-Ring1B (Cell Signalling #5694) "This antibody has been validated using SimpleChIP® Enzymatic Chromatin IP Kits." All other information can be found at https://www.cellsignal.com/products/primary-antibodies/ring1b-d22f2-rabbit-monoclonal-antibody/5694.
-Acetyl-Tubulin (Sigma-Aldrich #T7451) "Monoclonal Anti-Acetylated Tubulin antibody produced in mouse has been used in: quantitative dot blot, immunofluorescence, Western blot, immunocytochemistry, enzyme-linked immunosorbent assay (ELISA), solid-phase radioimmunoassay (RIA), electron microscopy" All other information can be found at https://www.sigmaaldrich.com/FR/en/product/sigma/t7451.
-Lamin B1 (abcam #ab16048) "KO validated for confirmed specificity." All other information can be found at: https://www.abcam.com/en-us/products/primary-antibodies/lamin-b1-antibody-nuclear-envelope-marker-ab16048.

In addition to external validations, we manually verified the profiles obtained in this study by comparing them with previous profiles obtained in our lab as well as published profiles of each of these marks, that were previously published using ES cells. All of the results were qualitatively comparable, both on loci that are known to be actively expressed in ES cells, on known Polycomb target genes such as Hox clusters, Wnt genes or Pax loci, as well as on known CTCF target sites or TAD boundary sites.

Eukaryotic cell lines

Policy information about cell lines and Sex and Gender in Research

Cell line source(s)	E14Tg2a.4 - purchased at MMRRC, donor is BayGenomics, BayGenomics Consortium, strain-129P2/OlaHsd.<br>CTCF-AID-eGFP E14Tga2 (ATCC, CRL-1821) published in Nora et al. (2017) DOI: 10.1016/j.molcel.2019.08.015 - gift from Ricardo Saldaña-Meyer.<br>PCGF4-/- PCGF2fl/fl Rosa26::CreERT2 ESCs published in Fursova et al. (2019) DOI: 10.1016/j.molcel.2019.03.024 - gift from Rob Klose.
Authentication	CTCF-AID-eGFP expression was confirmed by anti-GFP immunofluorescence. PCGF4 deletion and PCGF2 excision in response to tamoxifen (OHT) were verified by genotyping PCR.
Mycoplasma contamination	Cells were negative to mycoplasma spp. by PCR analysis.
Commonly misidentified lines<br>(See ICLAC register)	This study does not use any commonly misidentified lines.

Plants

Seed stocks	N/A
Novel plant genotypes	N/A
Authentication	N/A

ChIP-seq

Data deposition

☒ Confirm that both raw and final processed data have been deposited in a public database such as GEO.

☒ Confirm that you have deposited or provided access to graph files (e.g. BED files) for the called peaks.

Data access links<br>*May remain private before publication.*	Analysed and raw data are available under the GEO SuperSeries number GSE281151. ChIP-seq data files can be found under the GEO Series GSE280487.
Files in database submission	Bigwig files:<br>GSE280487_CTCF_mDMSO_dS.bam.bw<br>GSE280487_CTCF_mTSA.bam.bw

GSE280487_H3K9me3_m24hREC_dS.bam.bw
GSE280487_H3K9me3_mDMSO_dS.bam.bw
GSE280487_H3K9me3_mTSA_dS.bam.bw
GSE280487_K119Ub_mDMSO_scaled.bw
GSE280487_K119Ub_mREC_scaled.bw
GSE280487_K119Ub_mTSA_scaled.bw
GSE280487_K119Ub_mreREC_scaled.bw
GSE280487_K119Ub_mreTSA_scaled.bw
GSE280487_K27ac_mDMSO_scaled.bw
GSE280487_K27ac_mREC_scaled.bw
GSE280487_K27ac_mTSA_scaled.bw
GSE280487_K27ac_mreREC_scaled.bw
GSE280487_K27ac_mreTSA_scaled.bw
GSE280487_K27me3_mDMSO_scaled.bw
GSE280487_K27me3_mREC_scaled.bw
GSE280487_K27me3_mTSA_scaled.bw
GSE280487_K27me3_mreREC_scaled.bw
GSE280487_K27me3_mreTSA_scaled.bw
GSE280487_K4me1_mDMSO_scaled.bw
GSE280487_K4me1_mREC_scaled.bw
GSE280487_K4me1_mTSA_scaled.bw
GSE280487_K4me1_mreREC_scaled.bw
GSE280487_K4me1_mreTSA_scaled.bw
GSE280487_K4me3_mDMSO_scaled.bw
GSE280487_K4me3_mreREC_scaled.bw
GSE280487_K4me3_mreTSA_scaled.bw
GSE280487_K9ac_mDMSO_scaled.bw
GSE280487_K9ac_mREC_scaled.bw
GSE280487_K9ac_mTSA_scaled.bw
GSE280487_PCGF2_H2AK119Ub_mOHT_DMSO_scaled.bw
GSE280487_PCGF2_H2AK119Ub_mOHT_REC_scaled.bw
GSE280487_PCGF2_H2AK119Ub_mOHT_TSA_scaled.bw
GSE280487_PCGF2_H2AK119Ub_mUT_DMSO_scaled.bw
GSE280487_PCGF2_H3K27me3_mOHT_DMSO_scaled.bw
GSE280487_PCGF2_H3K27me3_mOHT_REC_scaled.bw
GSE280487_PCGF2_H3K27me3_mOHT_TSA_scaled.bw
GSE280487_PCGF2_H3K27me3_mUT_DMSO_scaled.bw
GSE280487_Ring1B_mDMSO_scaled.bw
GSE280487_Ring1B_mREC_scaled.bw
GSE280487_Ring1B_mTSA_scaled.bw
Peak files:
GSE280487_CTCF_DMSO_intersect.narrowPeak.gz
GSE280487_CTCF_TSA_intersect.narrowPeak.gz
GSE280487_H2AK119Ub_24hREC_intersect.broadPeak.gz
GSE280487_H2AK119Ub_DMSO_intersect.broadPeak.gz
GSE280487_H2AK119Ub_TSA_intersect.broadPeak.gz
GSE280487_H3K27ac_24hREC_intersect.broadPeak.gz
GSE280487_H3K27ac_DMSO_intersect.broadPeak.gz
GSE280487_H3K27ac_TSA_intersect.broadPeak.gz
GSE280487_H3K27me3_24hREC_intersect.broadPeak.gz
GSE280487_H3K27me3_DMSO_intersect.broadPeak.gz
GSE280487_H3K27me3_TSA_intersect.broadPeak.gz
GSE280487_H3K4me1_24hREC_intersect.broadPeak.gz
GSE280487_H3K4me1_TSA_intersect.broadPeak.gz
GSE280487_H3K4me3_24hREC_intersect.broadPeak.gz
GSE280487_H3K4me3_DMSO_intersect.broadPeak.gz
GSE280487_H3K4me3_TSA_intersect.broadPeak.gz
GSE280487_H3K9me3_24hREC_intersect.broadPeak.gz
GSE280487_H3K9me3_DMSO_intersect.broadPeak.gz
GSE280487_H3K9me3_TSA_intersect.broadPeak.gz
GSE280487_K119Ub_reREC_intersect.broadPeak.gz
GSE280487_K119Ub_reTSA_intersect.broadPeak.gz
GSE280487_K119ub_OHT_DMSO_intersect.broadPeak.gz
GSE280487_K119ub_OHT_REC_intersect.broadPeak.gz
GSE280487_K119ub_OHT_TSA_intersect.broadPeak.gz
GSE280487_K119ub_UT_DMSO_intersect.broadPeak.gz
GSE280487_K27ac_reREC_intersect.broadPeak.gz
GSE280487_K27ac_reTSA_intersect.broadPeak.gz
GSE280487_K27me3_OHT_DMSO_intersect.broadPeak.gz
GSE280487_K27me3_OHT_REC_intersect.broadPeak.gz
GSE280487_K27me3_OHT_TSA_intersect.broadPeak.gz
GSE280487_K27me3_UT_DMSO_intersect.broadPeak.gz
GSE280487_K27me3_mreTSA_scaled.bw
GSE280487_K27me3_reREC_intersect.broadPeak.gz
GSE280487_K27me3_reTSA_intersect.broadPeak.gz
GSE280487_K4me1_mreTSA_scaled.bw

GSE280487_K4me1_reREC_intersect.broadPeak.gz
GSE280487_K4me3_mreTSA_scaled.bw
GSE280487_K4me3_reREC_intersect.broadPeak.gz
GSE280487_K4me3_reTSA_intersect.broadPeak.gz
GSE280487_K9ac_DMSO_intersect.broadPeak.gz
GSE280487_K9ac_REC_intersect.broadPeak.gz
GSE280487_RAW.tar
GSE280487_Ring1B_DMSO_intersect.broadPeak.gz
GSE280487_Ring1B_REC_intersect.broadPeak.gz
Raw data files:
H3K4me1_DMSO_rep1_1.fq.gz
H3K4me1_DMSO_rep2_1.fq.gz
H3K4me1_TSA_rep1_1.fq.gz
H3K4me1_TSA_rep2_1.fq.gz
H3K4me1_24hREC_rep1_1.fq.gz
H3K4me1_24hREC_rep2_1.fq.gz
H3K4me3_DMSO_rep1_1.fq.gz
H3K4me3_DMSO_rep2_1.fq.gz
H3K4me3_TSA_rep1_1.fq.gz
H3K4me3_TSA_rep2_1.fq.gz
H3K4me3_24hREC_rep1_1.fq.gz
H3K4me3_24hREC_rep2_1.fq.gz
H3K9me3_DMSO_rep1_1.fq.gz
H3K9me3_DMSO_rep2_1.fq.gz
H3K9me3_TSA_rep1_1.fq.gz
H3K9me3_TSA_rep2_1.fq.gz
H3K9me3_24hREC_rep1_1.fq.gz
H3K9me3_24hREC_rep2_1.fq.gz
H3K27me3_DMSO_rep1_1.fq.gz
H3K27me3_DMSO_rep2_1.fq.gz
H3K27me3_TSA_rep1_1.fq.gz
H3K27me3_TSA_rep2_1.fq.gz
H3K27me3_24hREC_rep1_1.fq.gz
H3K27me3_24hREC_rep2_1.fq.gz
H3K27ac_DMSO_rep1_1.fq.gz
H3K27ac_DMSO_rep2_1.fq.gz
H3K27ac_TSA_rep1_1.fq.gz
H3K27ac_TSA_rep2_1.fq.gz
H3K27ac_24hREC_rep1_1.fq.gz
H3K27ac_24hREC_rep2_1.fq.gz
H2AK119Ub_DMSO_rep1_1.fq.gz
H2AK119Ub_DMSO_rep2_1.fq.gz
H2AK119Ub_TSA_rep1_1.fq.gz
H2AK119Ub_TSA_rep2_1.fq.gz
H2AK119Ub_24hREC_rep1_1.fq.gz
H2AK119Ub_24hREC_rep2_1.fq.gz
input_DMSO_rep1_1.fq.gz
input_DMSO_rep2_1.fq.gz
input_TSA_1.fq.gz
input_24hREC_1.fq.gz
H3K4me1_DMSO_rep1_2.fq.gz
H3K4me1_DMSO_rep2_2.fq.gz
H3K4me1_TSA_rep1_2.fq.gz
H3K4me1_TSA_rep2_2.fq.gz
H3K4me1_24hREC_rep1_2.fq.gz
H3K4me1_24hREC_rep2_2.fq.gz
H3K4me3_DMSO_rep1_2.fq.gz
H3K4me3_DMSO_rep2_2.fq.gz
H3K4me3_TSA_rep1_2.fq.gz
H3K4me3_TSA_rep2_2.fq.gz
H3K4me3_24hREC_rep1_2.fq.gz
H3K4me3_24hREC_rep2_2.fq.gz
H3K9me3_DMSO_rep1_2.fq.gz
H3K9me3_DMSO_rep2_2.fq.gz
H3K9me3_TSA_rep1_2.fq.gz
H3K9me3_TSA_rep2_2.fq.gz
H3K9me3_24hREC_rep1_2.fq.gz
H3K9me3_24hREC_rep2_2.fq.gz
H3K27me3_DMSO_rep1_2.fq.gz
H3K27me3_DMSO_rep2_2.fq.gz
H3K27me3_TSA_rep1_2.fq.gz
H3K27me3_TSA_rep2_2.fq.gz
H3K27me3_24hREC_rep1_2.fq.gz
H3K27me3_24hREC_rep2_2.fq.gz
H3K27ac_DMSO_rep1_2.fq.gz
H3K27ac_DMSO_rep2_2.fq.gz

H3K27ac_TSA_rep1_2.fq.gz
H3K27ac_TSA_rep2_2.fq.gz
H3K27ac_24hREC_rep1_2.fq.gz
H3K27ac_24hREC_rep2_2.fq.gz
H2AK119Ub_DMSO_rep1_2.fq.gz
H2AK119Ub_DMSO_rep2_2.fq.gz
H2AK119Ub_TSA_rep1_2.fq.gz
H2AK119Ub_TSA_rep2_2.fq.gz
H2AK119Ub_24hREC_rep1_2.fq.gz
H2AK119Ub_24hREC_rep2_2.fq.gz
input_DMSO_rep1_2.fq.gz
input_DMSO_rep2_2.fq.gz
input_TSA_2.fq.gz
input_24hREC_2.fq.gz

H3K27ac_reTSA_rep1_1.fq.gz
H3K27ac_reTSA_rep2_1.fq.gz
H3K4me1_reTSA_rep1_1.fq.gz
H3K4me1_reTSA_rep2_1.fq.gz
H3K4me3_reTSA_rep1_1.fq.gz
H3K4me3_reTSA_rep2_1.fq.gz
H3K27me3_reTSA_rep1_1.fq.gz
H3K2ume3_reTSA_rep2_1.fq.gz
H2AK119Ub_reTSA_rep1_1.fq.gz
H2AK119Ub_reTSA_rep2_1.fq.gz
input_reTSA_rep1_1.fq.gz
input_reTSA_rep2_1.fq.gz
H3K27ac_reREC_rep1_1.fq.gz
H3K27ac_reREC_rep2_1.fq.gz
H3K4me1_reREC_rep1_1.fq.gz
H3K4me1_reREC_rep2_1.fq.gz
H3K4me3_reREC_rep1_1.fq.gz
H3K4me3_reREC_rep2_1.fq.gz
H3K27me3_reREC_rep1_1.fq.gz
H3K2ume3_reREC_rep2_1.fq.gz
H2AK119Ub_reREC_rep1_1.fq.gz
H2AK119Ub_reREC_rep2_1.fq.gz
input_reREC_rep1_1.fq.gz
input_reREC_rep2_1.fq.gz
H3K9ac_DMSO_rep3_1.fq.gz
H3K9ac_DMSO_rep4_1.fq.gz
Ring1B_DMSO_rep3_1.fq.gz
Ring1B_DMSO_rep4_1.fq.gz
H3K27ac_DMSO_rep3_1.fq.gz
H3K4me1_DMSO_rep4_1.fq.gz
input_DMSO_rep3_1.fq.gz
input_DMSO_rep4_1.fq.gz
H3K9ac_TSA_rep3_1.fq.gz
H3K9ac_TSA_rep4_1.fq.gz
Ring1B_TSA_rep3_1.fq.gz
Ring1B_TSA_rep4_1.fq.gz
H3K27ac_TSA_rep3_1.fq.gz
input_TSA_rep3_1.fq.gz
input_TSA_rep4_1.fq.gz
H3K9ac_REC_rep3_1.fq.gz
H3K9ac_REC_rep4_1.fq.gz
Ring1B_REC_rep3_1.fq.gz
Ring1B_REC_rep4_1.fq.gz
H3K27ac_REC_rep3_1.fq.gz
input_REC_rep3_1.fq.gz
input_REC_rep4_1.fq.gz
TSA_YY1_rep1_1.fq.gz
TSA_YY1_L1_rep2_1.fq.gz
TSA_input_rep1_1.fq.gz
H3K27me3_PCGF2_UT_DMSO_rep1_1.fq.gz
H3K27me3_PCGF2_UT_DMSO_rep2_1.fq.gz
H2AK119Ub_PCGF2_UT_DMSO_rep1_1.fq.gz
H2AK119Ub_PCGF2_UT_DMSO_rep2_1.fq.gz
input_PCGF2_UT_DMSO_1.fq.gz
H3K27me3_PCGF2_OHT_DMSO_rep1_1.fq.gz
H3K27me3_PCGF2_OHT_DMSO_rep2_1.fq.gz
H2AK119Ub_PCGF2_OHT_DMSO_rep1_1.fq.gz
H2AK119Ub_PCGF2_OHT_DMSO_rep2_1.fq.gz
input_PCGF2_OHT_DMSO_1.fq.gz
H3K27me3_PCGF2_OHT_TSA_rep1_1.fq.gz
H3K27me3_PCGF2_OHT_TSA_rep2_1.fq.gz

H2AK119Ub_PCGF2_OHT_TSA_rep1_1.fq.gz
H2AK119Ub_PCGF2_OHT_TSA_rep2_1.fq.gz
input_PCGF2_OHT_TSA_1.fq.gz
H3K27me3_PCGF2_OHT_REC_rep1_1.fq.gz
H3K27me3_PCGF2_OHT_REC_rep2_1.fq.gz
H2AK119Ub_PCGF2_OHT_REC_rep1_1.fq.gz
H2AK119Ub_PCGF2_OHT_REC_rep2_1.fq.gz
input_PCGF2_OHT_REC_1.fq.gz
H3K27ac_reTSA_rep1_2.fq.gz
H3K27ac_reTSA_rep2_2.fq.gz
H3K4me1_reTSA_rep1_2.fq.gz
H3K4me1_reTSA_rep2_2.fq.gz
H3K4me3_reTSA_rep1_2.fq.gz
H3K4me3_reTSA_rep2_2.fq.gz
H3K27me3_reTSA_rep1_2.fq.gz
H3K2ume3_reTSA_rep2_2.fq.gz
H2AK119Ub_reTSA_rep1_2.fq.gz
H2AK119Ub_reTSA_rep2_2.fq.gz
input_reTSA_rep1_2.fq.gz
input_reTSA_rep2_2.fq.gz
H3K27ac_reREC_rep1_2.fq.gz
H3K27ac_reREC_rep2_2.fq.gz
H3K4me1_reREC_rep1_2.fq.gz
H3K4me1_reREC_rep2_2.fq.gz
H3K4me3_reREC_rep1_2.fq.gz
H3K4me3_reREC_rep2_2.fq.gz
H3K27me3_reREC_rep1_2.fq.gz
H3K2ume3_reREC_rep2_2.fq.gz
H2AK119Ub_reREC_rep1_2.fq.gz
H2AK119Ub_reREC_rep2_2.fq.gz
input_reREC_rep1_2.fq.gz
input_reREC_rep2_2.fq.gz
H3K9ac_DMSO_rep3_2.fq.gz
H3K9ac_DMSO_rep4_2.fq.gz
Ring1B_DMSO_rep3_2.fq.gz
Ring1B_DMSO_rep4_2.fq.gz
H3K27ac_DMSO_rep3_2.fq.gz
H3K4me1_DMSO_rep4_2.fq.gz
input_DMSO_rep3_2.fq.gz
input_DMSO_rep4_2.fq.gz
H3K9ac_TSA_rep3_2.fq.gz
H3K9ac_TSA_rep4_2.fq.gz
Ring1B_TSA_rep3_2.fq.gz
Ring1B_TSA_rep4_2.fq.gz
H3K27ac_TSA_rep3_2.fq.gz
input_TSA_rep3_2.fq.gz
input_TSA_rep4_2.fq.gz
H3K9ac_REC_rep3_2.fq.gz
H3K9ac_REC_rep4_2.fq.gz
Ring1B_REC_rep3_2.fq.gz
Ring1B_REC_rep4_2.fq.gz
H3K27ac_REC_rep3_2.fq.gz
input_REC_rep3_2.fq.gz
input_REC_rep4_2.fq.gz
TSA_YY1_rep1_2.fq.gz
TSA_YY1_L1_rep2_2.fq.gz
TSA_input_rep1_2.fq.gz
H3K27me3_PCGF2_UT_DMSO_rep1_2.fq.gz
H3K27me3_PCGF2_UT_DMSO_rep2_2.fq.gz
H2AK119Ub_PCGF2_UT_DMSO_rep1_2.fq.gz
H2AK119Ub_PCGF2_UT_DMSO_rep2_2.fq.gz
input_PCGF2_UT_DMSO_2.fq.gz
H3K27me3_PCGF2_OHT_DMSO_rep1_2.fq.gz
H3K27me3_PCGF2_OHT_DMSO_rep2_2.fq.gz
H2AK119Ub_PCGF2_OHT_DMSO_rep1_2.fq.gz
H2AK119Ub_PCGF2_OHT_DMSO_rep2_2.fq.gz
input_PCGF2_OHT_DMSO_2.fq.gz
H3K27me3_PCGF2_OHT_TSA_rep1_2.fq.gz
H3K27me3_PCGF2_OHT_TSA_rep2_2.fq.gz
H2AK119Ub_PCGF2_OHT_TSA_rep1_2.fq.gz
H2AK119Ub_PCGF2_OHT_TSA_rep2_2.fq.gz
input_PCGF2_OHT_TSA_2.fq.gz
H3K27me3_PCGF2_OHT_REC_rep1_2.fq.gz
H3K27me3_PCGF2_OHT_REC_rep2_2.fq.gz
H2AK119Ub_PCGF2_OHT_REC_rep1_2.fq.gz

H2AK119Ub_PCGF2_OHT_REC_rep2_2.fq.gz
input_PCGF2_OHT_REC_2.fq.gz

Genome browser session
(e.g. UCSC)

IGV

Methodology

Replicates

All ChIP-seq experiments were done in biological duplicates, H3K27ac ChIP-seq was done in biological triplicates.

Sequencing depth

Epitope Condition Replicate Uniquely mapped reads
H3K4me1 TSA 1 54818314
H3K4me3 TSA 1 68009818
H3K9me3 TSA 1 33008412
H3K27me3 TSA 1 57480252
H3K27ac TSA 1 78013842
H2AK119ub TSA 1 61046204
CTCF TSA 1 54263736
YY1 TSA 1 26802614
YY1 DMSO 1 19484622
input TSA 1 19133166
H3K4me1 TSA 2 61371056
H3K4me3 TSA 2 65808876
H3K9me3 TSA 2 48287118
H3K27me3 TSA 2 55112334
H3K27ac TSA 2 65446160
H2AK119ub TSA 2 48330640
Rad21 TSA 2 54363204
CTCF TSA 2 60778948
YY1 TSA 2 31665080
YY1 DMSO 2 35199046
input DMSO 1 16692324
H3K4me1 DMSO 1 45412782
H3K4me3 DMSO 1 43890218
H3K9me3 DMSO 1 28781034
H3K27me3 DMSO 1 46382548
H3K27ac DMSO 1 44150428
H2AK119ub DMSO 1 37625772
Rad21 DMSO 1 47885902
CTCF DMSO 1 44213344
H3K4me1 24hREC 1 71991732
H3K4me1 24hREC 1 73380982
H3K9me3 24hREC 1 22442310
H3K27me3 24hREC 1 35979550
H3K27ac 24hREC 1 85291528
H2AK119ub 24hREC 1 28678806
H3K4me1 24hREC 2 65286048
H3K4me3 24hREC 2 64448520
H3K9me3 24hREC 2 34203612
H3K27me3 24hREC 2 28833974
H3K27ac 24hREC 2 75452030
H2AK119ub 24hREC 2 53702010
H3K4me1 DMSO 2 60862026
H3K4me3 DMSO 2 66707678
H3K9me3 DMSO 2 24801870
H3K27me3 DMSO 2 35094396
H3K27ac DMSO 2 72168012
H2AK119ub DMSO 2 22036164
Rad21 DMSO 2 69194288
CTCF DMSO 2 64134764
input 24hREC 1 25297596
input DMSO 2 21755478
H3K27ac reTSA 1 68176590
H3K4me1 reTSA 1 46840426
H3K4me3 reTSA 1 55506436
H3K27me3 reTSA 1 69242556
H2AK119Ub reTSA 1 57296340
H3K27ac reTSA 2 71278248
H3K4me1 reTSA 2 54732654
H3K4me3 reTSA 2 61073612
H3K27me3 reTSA 2 60488472
H2AK119Ub reTSA 2 49177130
input reTSA 1 25688120
input reTSA 2 26598012
H3K27ac reREC 1 54721142
H3K4me1 reREC 1 49971722

H3K4me3 reREC 1 59984504
H3K27me3 reREC 1 59279822
H2AK119Ub reREC 1 60537406
H3K27ac reREC 2 61175514
H3K4me1 reREC 2 51603532
H3K4me3 reREC 2 58975348
H3K27me3 reREC 2 57988216
H2AK119Ub reREC 2 69468460
input reREC 1 20212252
input reREC 2 23208486
H3K27ac DMSO 3 53906164
H3K9ac DMSO 3 38367614
Ring1B DMSO 3 49224398
H3K9ac DMSO 4 38150380
Ring1B DMSO 4 50305330
H3K4me1 DMSO 4 46965044
H3K27ac TSA 3 66816874
H3K9ac TSA 3 58712350
Ring1B TSA 3 58902498
H3K9ac TSA 4 54175406
Ring1B TSA 4 59741032
H3K27ac REC 3 49171736
H3K9ac REC 3 52634916
Ring1B REC 3 57112740
H3K9ac REC 4 30452666
Ring1B REC 4 50993230
input DMSO 3 34464872
input DMSO 4 33952542
input TSA 3 32588284
input TSA 4 35452042
input REC 3 33429800
input REC 4 21573896
H3K27me3 PCGF2 -OHT +DMSO 1 46259940
H3K27me3 PCGF2 -OHT +DMSO 2 50642716
H2AK119Ub PCGF2 -OHT +DMSO 1 44492246
H2AK119Ub PCGF2 -OHT +DMSO 2 50212746
input PCGF2 -OHT +DMSO 1 33166120
H3K27me3 PCGF2 +OHT +DMSO 1 38362120
H3K27me3 PCGF2 +OHT +DMSO 2 47164752
H2AK119Ub PCGF2 +OHT +DMSO 1 51489588
H2AK119Ub PCGF2 +OHT +DMSO 2 44400782
input PCGF2 +OHT +DMSO 1 36482660
H3K27me3 PCGF2 +OHT +TSA 1 56949378
H3K27me3 PCGF2 +OHT +TSA 2 53889352
H2AK119Ub PCGF2 +OHT +TSA 1 47727028
H2AK119Ub PCGF2 +OHT +TSA 2 46566516
input PCGF2 +OHT +TSA 1 22281632
H3K27me3 PCGF2 +OHT +REC 1 54817788
H3K27me3 PCGF2 +OHT +REC 2 57616746
H2AK119Ub PCGF2 +OHT +REC 1 54138000
H2AK119Ub PCGF2 +OHT +REC 2 53981734
input PCGF2 +OHT +REC 1 36046280

Antibodies

The following antibodies were used:
- H3K4me1 (ActiveMotif #39297)
- H3K4me3 (Millipore #04-745)
- H3K9me3 (abcam #8898)
- H3K27me3 (ActiveMotif #39155)
- H3K27Ac (ActiveMotif #39133)
- H2AK119Ub (Cell Signalling #8240S)
- CTCF (Active Motif #61311)
- YY1 (abcam #109237)
- H3K9Ac (Millipore #07-352)
- Ring1B (Cell Signalling #5694)

Peak calling parameters

We called peaks with MACS3 (https://hbctraining.github.io/Intro-to-ChIPseq-flipped/lessons/06_peak_calling_macs.html) with default setting; for histone marks with the --broad option specified. More detailed in the Methods.

Data quality

Assessed by reproducibility of published data.

Software

As described in the Methods ChIP-seq samples were mapped using bowtie2 v.2.3.5.1 (https://bowtie-bio.sourceforge.net/bowtie2/index.shtml) with command “bowtie2 -p 12 --no-mixed --no-discordant” 65. Then, we used samtools v.1.9 (https://www.htslib.org/doc/samtools-view.html) to filter out low-quality reads (command “samtools view -b -q 30“). Finally, we used Sambamba v1.0

(https://github.com/biod/sambamba) to sort the bam files (command “sambamba sort”), deduplicate, and index them (“sambamba markdup --remove-duplicates”) with default parameters.

Flow Cytometry

Plots

Confirm that:

☒ The axis labels state the marker and fluorochrome used (e.g. CD4-FITC).

☒ The axis scales are clearly visible. Include numbers along axes only for bottom left plot of group (a 'group' is an analysis of identical markers).

☒ All plots are contour plots with outliers or pseudocolor plots.

☒ A numerical value for number of cells or percentage (with statistics) is provided.

Methodology

Sample preparation	1-3x106 mESCs were dissociated with TrypLE, pelleted, and resuspended in PBS. For cell cycle analysis, dissociated mESCs were washed once in PBS and pelleted and fixed in cold 70% ethanol for 30 min at 4°C. Cells were stained with the Propidium Iodide Flow Cytometry Kit (Abcam #ab139418) according to manufacturer’s instruction. Flow cytometry was performed on a CytoFlex instrument using CytExpert (v2.4), and analysis was performed using the FlowJo (v10.10) software. For cell proliferation tracing, dissociated mESCs were stained with 1 µM CellTrace Violet staining solution (Invitrogen #C34571) according to manufacturer’s instructions, and were plated on gelatine-coated cell culture dishes. After 24 hours, TSA treatment and washes were performed as described before and cells were harvested following a further 24-hour incubation period. Collected cells were fixed in 4% PFA for 10 minutes at room temperature, washed with PBS and preserved at 4°C until further use. Flow cytometry was performed on a Novocyte Quanteon instrument, and analysis was performed using the NovoExpress (v1.6.3) software.
Instrument	CytoFlex (Beckman) or Novocyte Quanteon (Agilent)
Software	CytExpert (v2.4)<br>NovoExpress (v1.6.3)<br>FlowJo (v10.10)
Cell population abundance	20000-30000 cells were assayed for each sample.
Gating strategy	Gating was based on the pattern of FSC-A/SSC-A. Singlets were gated based on the pattern of FSC-H/FSC-A.

☒ Tick this box to confirm that a figure exemplifying the gating strategy is provided in the Supplementary Information.
